# Reliability of Acromiohumeral Interval Measurement on Nonstandardized Shoulder Radiographs by Reporting Radiographers: A Retrospective Service Evaluation

**DOI:** 10.7759/cureus.111016

**Published:** 2026-06-17

**Authors:** Tien Parrymore-Peers

**Affiliations:** 1 Department of Radiology, University Hospitals of Leicester NHS Trust, Leicester, GBR

**Keywords:** acromiohumeral interval (ahi), full-thickness rotator cuff tear, intraclass correlation coefficient (icc), measurement reliability, reporting radiographer, rotator cuff tear, shoulder radiograph

## Abstract

Introduction: The acromiohumeral interval (AHI) on shoulder radiographs is widely used as an indirect marker of chronic rotator cuff (RC) pathology, but routine anteroposterior (AP) radiographs are often nonstandardized, and reliability in day-to-day reporting is uncertain. This service evaluation assessed inter- and intraobserver reliability of AHI measurement by reporting radiographers and examined correspondence with ultrasound for identifying clinically significant RC tears.

Methods: Seventy-three nonstandardized AP shoulder radiographs from a UK NHS Trust atraumatic shoulder pathway (age 18-70 years) with a Trust-performed shoulder ultrasound report within six months were independently assessed by three reporting radiographers. Each observer measured AHI (mm, one decimal place) and recorded the Hamada grade; measurements were repeated after two weeks. Reliability was assessed using intraclass correlation coefficients (ICCs). Measurement error was quantified using the standard error of measurement (SEM) and the minimal detectable change at 95% confidence (MDC95). Ultrasound reports were dichotomized as positive for full-thickness (complete) or massive RC tear and negative for partial-thickness tear, tendinopathy, or no tear. Radiographs were dichotomized as AHI ≤6.0 mm (positive) vs. >6.0 mm (negative).

Results: Intraobserver reliability was good to excellent (ICC, 0.88-0.98 (good to excellent)). Interobserver reliability was excellent in Trial 1 (ICC, 0.95 (excellent)) and good to excellent in Trial 2 (ICC, 0.92 (good to excellent)). SEM was <1 mm, and MDC95 was approximately 1.6-2.4 mm. The Hamada agreement was weak to moderate. Using the definitions above, AHI ≤6.0 mm showed a mean sensitivity of 42% and a mean specificity of 94% (mean across observers and trials), with an overall accuracy above 70%.

Conclusions: Reporting radiographers measured AHI on nonstandardized AP radiographs with good-to-excellent reliability. Although sensitivity is limited (as expected for an indirect marker of advanced disease), high specificity supports AHI ≤6.0 mm as a rule-in feature for chronic full-thickness or massive RC tears. Standardized landmark guidance and focused training on common pitfalls (arm abduction, os acromiale) may further improve consistency.

## Introduction

Shoulder pain is the third most common musculoskeletal condition presenting to general practitioners (GPs), after lower back and knee pain, with around 1% of adults consulting a GP each year [1]. The most common causes include rotator cuff (RC) disease, adhesive capsulitis, and osteoarthritis. RC disease accounts for approximately 65%-70% of shoulder pain presentations [2]. RC pathology is associated with substantial disability and reduced quality of life; quality-of-life impairment has been reported to be comparable to chronic systemic conditions such as congestive heart failure and diabetes [3].

The RC comprises four tendons (supraspinatus, infraspinatus, teres minor, subscapularis) that provide dynamic stability and controlled rotational movement of the humerus [4]. RC pathology may arise from trauma, acute overload, or progressive degeneration. Degenerative RC disease may remain asymptomatic until an increase in load or gradual tear progression causes pain and functional impairment. When RC force coupling is disrupted, superior migration of the humeral head can occur, narrowing the acromiohumeral interval (AHI), the shortest distance between the inferior acromial cortex and the humeral head. The AHI region contains the RC tendons and subacromial-subdeltoid bursa and is therefore biologically linked to the consequences of cuff failure [4].

Radiographs are recommended as first-line imaging in atraumatic shoulder pain to exclude red flags, such as fracture, aggressive osseous lesions, infection, and advanced arthropathy, and they may demonstrate indirect signs of chronic RC disease [5,6]. A markedly reduced AHI, most commonly defined as 6 mm or less, has long been considered strongly indicative of chronic full-thickness or massive RC tears [7,8]. Orthopedic surgeons also use AHI measurements to contextualize outcomes after surgery and to support the classification of cuff tear arthropathy [9,10]. However, in routine practice, AHI measurement can be affected by patient positioning and radiographic technique. Experimental and clinical studies have shown that arm abduction and beam orientation can alter the apparent AHI by several millimeters, which may be clinically meaningful when applying a fixed diagnostic threshold [11,12].

Clinical practice guidelines (CPGs) for nontraumatic shoulder pain recommend an initial period of conservative management, typically physiotherapy, with radiographs undertaken if symptoms persist or red flags are suspected; routine advanced imaging in primary care is commonly discouraged [1,5,6,13-15]. In many NHS pathways, access to ultrasound may be constrained, and waiting times can delay definitive diagnosis. Although some guidance acknowledges that a reduced AHI is consistent with chronic RC disease, radiographic findings do not always trigger pathway changes [15]. This creates a practical question for services using radiographer-led reporting: can AHI be measured reliably on routine, nonstandardized radiographs, and when reduced, how well does it correspond to ultrasound-confirmed full-thickness or massive tears?

At the authors' Trust, patients under 70 years with chronic atraumatic shoulder pain referred by GPs undergo anteroposterior (AP) shoulder radiographs as part of a standard pathway. Where radiographs do not identify a clear cause, ultrasound may be requested for RC assessment. Reporting radiographers already comment on AHI in their reports, but findings do not currently alter subsequent triage. While studies on AHI reliability exist, many use standardized projections or exclude technically suboptimal images [16-19]. There is limited published evidence evaluating AHI measurement reliability specifically among reporting radiographers working under routine clinical reporting conditions using nonstandardized radiographs typical of day-to-day practice [16-19]. This service evaluation, therefore, examined the reliability and clinical correspondence of AHI measurement performed by reporting radiographers on nonstandardized AP radiographs.

This study aimed to evaluate whether AHI measurement on nonstandardized AP shoulder radiographs can be used to identify clinically significant RC tears. The objectives are to 1) evaluate the intraobserver reliability of reporting radiographers measuring AHI, 2) evaluate the interobserver reliability of reporting radiographers measuring AHI, and 3) evaluate whether AHI ≤6.0 mm corresponds with ultrasound-confirmed full-thickness or massive RC tear.

This work was previously presented as an e-poster at the UK Imaging and Oncology Congress in June 2024 and as an oral presentation at the European Congress of Radiology in March 2025.

## Materials and methods

Study design

This was a retrospective service evaluation conducted within an NHS tertiary teaching Trust. The study was reported with reference to the Guidelines for Reporting Reliability and Agreement Studies [20] and the Quality Appraisal of Reliability Studies [21].

Population and eligibility criteria

The sampling frame comprised patients imaged under the Trust atraumatic shoulder pathway between January 1 and December 31, 2021. Inclusion criteria were 1) GP referral for nontraumatic shoulder pain, 2) age 18-70 years, 3) an AP shoulder radiograph available, and 4) a Trust-performed shoulder ultrasound report within six months of the radiograph. The upper age limit reflected local pathway criteria and reduced confounding from advanced degenerative change and cuff tear arthropathy, which become increasingly prevalent with age and may influence AHI independently of pathway-relevant RC tears [22].

Sampling and sample size

A total of 1,091 eligible cases were identified. A finite population sample size calculation using a 95% confidence level (Z = 1.96) and 5% margin of error yielded a target sample of 75. Two cases were excluded after review because of trauma occurring between the two imaging episodes within the six-month interval, leaving 73 radiographs for analysis. Simple random sampling was performed by assigning each eligible case a number and selecting cases using a random number generator. The same set of radiographs was used in Trial 2, with order rerandomized.

Observers

Eligible observers were reporting radiographers authorized to provide definitive musculoskeletal reports for GP-referred radiographs. At the authors’ Trust, seven reporting radiographers met the eligibility criteria; three were randomly selected from those who consented to participate. The selected observers had four, seven, and 18 years of reporting experience, reflecting the experience spectrum of the service. Written consent was obtained from the participating reporting radiographers, who are referenced only as Observers 1-3 to preserve anonymity.

Reference standard and index test (diagnostic definitions)

Ultrasound (Reference Standard)

All ultrasounds were performed by Trust sonographers as part of routine clinical care. Ultrasound reports were dichotomized as positive for a full-thickness (complete) or massive RC tear and negative for partial-thickness tear, tendinopathy, or no tear. If a report used probabilistic language (e.g., “probable partial-thickness tear”) without stating a definite full-thickness tear, it was classified as partial-thickness (negative) for the primary analysis. If a report included both a “suspected” full-thickness tear of one tendon and a definite full-thickness tear of another tendon, the case was classified as ultrasound-positive.

Radiograph (Index Test)

Radiographs were dichotomized as positive if AHI ≤6.0 mm and negative if AHI >6.0 mm.

Blinding and measurement procedure

Radiographs were anonymized prior to review. Observers were blinded to patient demographics, clinical history, prior imaging, ultrasound results, and each other’s readings to reduce interpretation bias [21]. Radiographs were reviewed on standard reporting workstations in the observers’ usual reporting environment to reflect routine practice.

**Figure 1:**
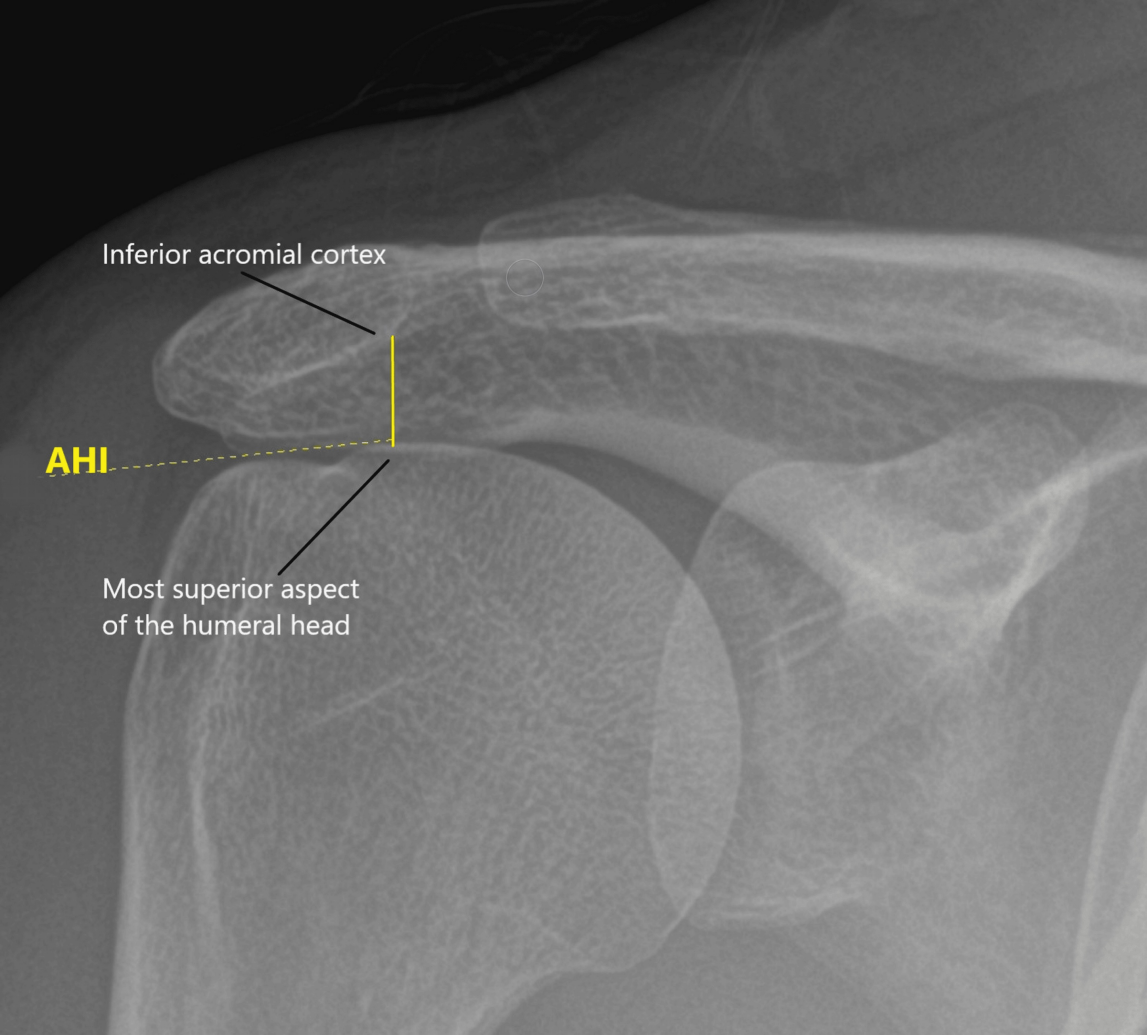
Measuring the AHI on an AP shoulder radiograph An annotated deidentified AP shoulder radiograph demonstrating the AHI measurement method used in this evaluation. AHI is defined as the shortest distance from the inferior acromial cortex to the most superior aspect of the humeral head, measured to one decimal place AHI: acromiohumeral interval; AP: anteroposterior

Observers measured the AHI in millimeters to one decimal place as the shortest distance from the inferior acromial cortex to the most superior aspect of the humeral head, consistent with radiographic definitions used in prior work (Figure 1) [16]. Hamada classification was recorded to reflect clinical communication with orthopedics and to explore agreement for an established radiographic grading system of cuff tear arthropathy [9,10]; a summary of the grading criteria is provided in the Appendix.

Because the evaluation aimed to reflect routine reporting conditions, radiographs were not excluded on the basis of positioning or projection variability; the potential impact of nonstandardized acquisition on landmark selection and measurement variability is explored in the Results and Discussion section.

No additional training was provided for AHI measurement, as it is already part of routine reporting practice. A brief reference sheet describing Hamada grading was provided because it was not routinely used in the service.

To minimize recall bias for intraobserver testing between Trial 1 and Trial 2, a two-week washout interval was used. Trial 2 used the same images in a different randomized order.

Statistical analysis

Continuous AHI measurements were analyzed using intraclass correlation coefficients (ICCs) because the aim was agreement rather than correlation [23]. Intraobserver ICCs were calculated using a two-way mixed-effects model with absolute agreement; interobserver ICCs were calculated using a two-way random-effects model to support generalization to reporting radiographers in the service [23,24]. ICC values were interpreted using the thresholds proposed by Koo and Li: <0.50 poor, 0.50-0.75 moderate, 0.75-0.90 good, and >0.90 excellent [24].

Because ICC does not express measurement error in units, standard error of measurement (SEM) and minimal detectable change at 95% confidence (MDC95) were calculated to quantify the expected error in millimeters and the smallest change exceeding error, respectively [25,26]. SEM was calculated as pooled $\mathrm{SEM} = \mathrm{SD} \times \sqrt{1 - \mathrm{ICC}}$ and MDC95 as $\mathrm{MDC}_{95} = 1.96 \times \sqrt{2} \times \mathrm{SEM}$ [25,26].

Diagnostic validity of AHI ≤6.0 mm for identifying ultrasound-confirmed full-thickness or massive RC tear was assessed using sensitivity, specificity, accuracy, positive predictive value (PPV), and negative predictive value (NPV). These diagnostic metrics were calculated separately for each observer and trial, then summarized as an unweighted mean across observers and trials. Categorical agreement for Hamada grades was assessed using kappa statistics [27].

## Results

Sample characteristics

The sample included 73 radiographs with paired ultrasound reports. Demographics were not analyzed further because age was restricted to 18-70 years, and additional demographic variables were not required to address the aims.

Intraobserver reliability

Intraobserver reliability for AHI measurement was good to excellent, with ICCs ranging from 0.88 to 0.98 (Table 1).

**Table 1:** Intraobserver reliability of AHI measurement ICC interpretation uses Koo and Li thresholds ICC: intraclass correlation coefficient; CI: confidence interval; SEM: standard error of measurement; MDC95: minimal detectable change at 95% confidence; AHI: acromiohumeral interval

Observer	ICC	95% CI	Interpretation	SEM (mm)	MDC95 (mm)
1	0.88	0.82-0.93	Good	0.76	2.42
2	0.98	0.96-0.99	Excellent	0.34	1.62
3	0.98	0.96-0.99	Excellent	0.40	1.76

**Figure 2:**
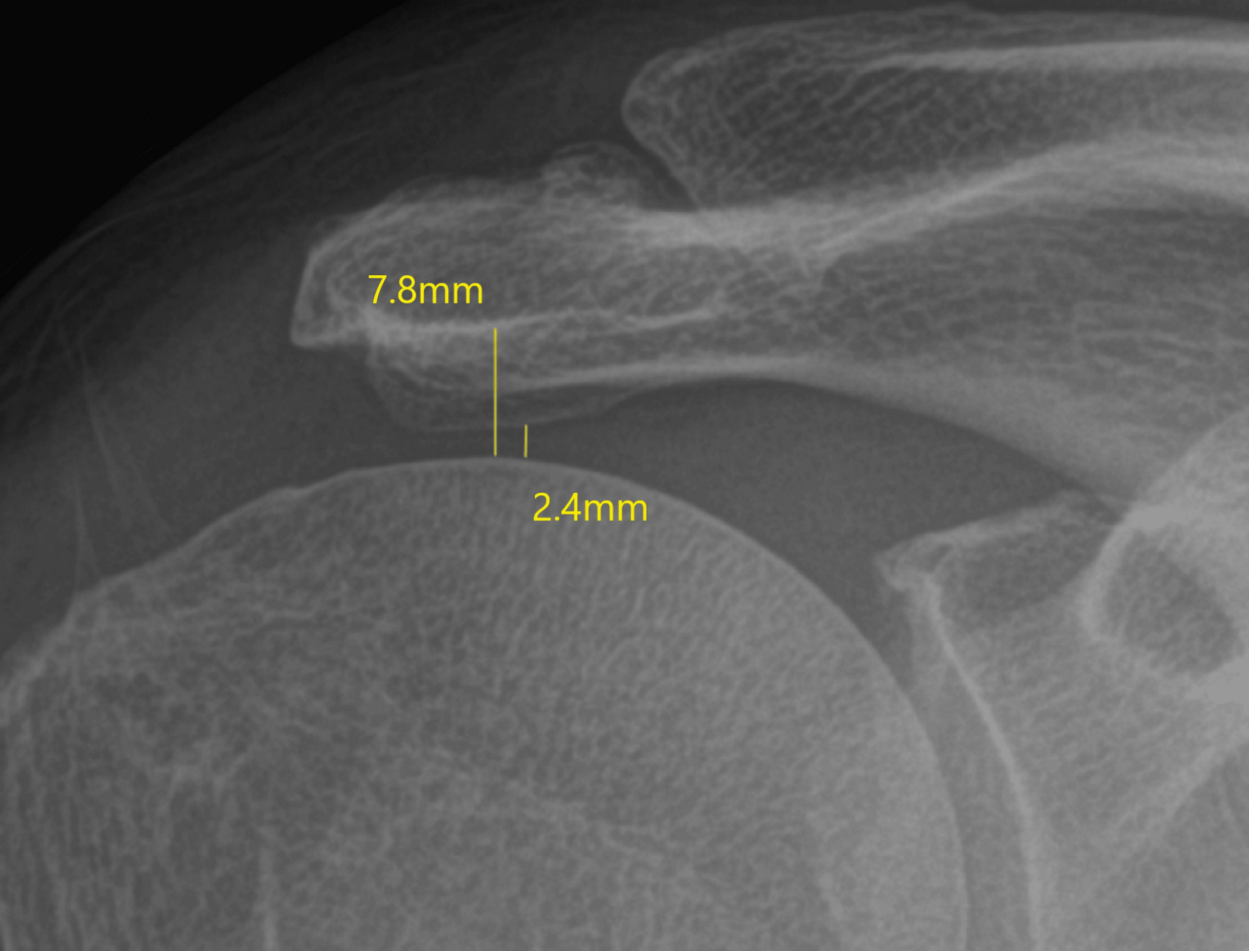
Maximal intraobserver AHI discrepancy due to inferior acromial landmark selection Radiograph illustrating the greatest intraobserver measurement discrepancy (Observer 1) due to inferior acromial landmark selection ambiguity on a nonstandardized AP view. Both measurements are shown on the same image: Trial 1 measured 7.8 mm using the inferior acromial/subacromial contour as the reference point, whereas Trial 2 measured 2.4 mm when an alternative inferior adjacent bony margin was selected, producing a clinically meaningful threshold-crossing difference AHI: acromiohumeral interval​​​​​​​; AP: anteroposterior​​​​​​​

Differences exceeding MDC95 occurred in 6/73 (8.2%), 4/73 (5.5%), and 3/73 (4.1%) observations for Observers 1-3, respectively, with a maximum within-observer difference of 5.36 mm (Table 2). The largest within intraobserver difference (Observer 1) reflected ambiguity in selecting the inferior acromial reference point, as illustrated in Figure 2.

**Table 2:** Intraobserver differences exceeding MDC95 Percentages are out of 73 radiographs. Max absolute difference is the greatest within-observer difference between Trial 1 and Trial 2 MDC95: minimal detectable change at 95% confidence

Observer	MDC95 (mm)	n > MDC95, n (%)	Max absolute difference (mm)
1	2.42	6 (8.2%)	5.36
2	1.62	4 (5.5%)	2.00
3	1.76	3 (4.1%)	3.91

Interobserver reliability

Interobserver reliability was excellent in Trial 1 (ICC 0.95) and good to excellent in Trial 2 (ICC 0.92), with SEM values of 0.53 and 0.68 mm and MDC95 values of 2.02 and 2.29 mm, respectively (Table 3).

**Table 3:** Interobserver reliability of AHI measurement Trial 1 and Trial 2 refer to the two trials. ICC interpretation uses Koo and Li thresholds ICC: intraclass correlation coefficient; CI: confidence interval; SEM: standard error of measurement; MDC95: minimal detectable change at 95% confidence; AHI: acromiohumeral interval

Trial	ICC	95% CI	Interpretation	SEM (mm)	MDC95 (mm)
1	0.95	0.93-0.97	Excellent	0.53	2.02
2	0.92	0.88-0.95	Good-excellent	0.68	2.29

Between-observer ranges exceeding MDC95 occurred in 13/73 (17.8%) observations in Trial 1 and 15/73 (20.5%) in Trial 2, with maximum between-observer differences of 6.17 and 6.83 mm, respectively (Table 4).

**Table 4:** Interobserver differences exceeding MDC95 Percentages are out of 73 radiographs. Max absolute difference is the greatest between-observer difference within each trial MDC95: minimal detectable change at 95% confidence

Trial	MDC95 (mm)	n > MDC95, n (%)	Max absolute difference (mm)
1	2.02	13 (17.8%)	6.17
2	2.29	15 (20.5%)	6.83

These maximum values represent the single greatest between-observer range within each trial and occurred in a subset of technically challenging, nonstandardized radiographs. Importantly, overall interobserver measurement error remained low (SEM 0.53-0.68 mm; MDC95 2.02-2.29 mm), indicating that most measurements clustered closely, while the largest differences reflect outliers related to positioning/projection and inferior landmark selection.

Diagnostic accuracy vs. ultrasound

Using ultrasound as the reference standard, AHI ≤6.0 mm demonstrated low sensitivity and high specificity across observers and trials (Table 5). Ultrasound prevalence of full-thickness/massive tear was 38%, while the radiograph positivity rate (AHI ≤6.0 mm) varied by observer and trial (Table 6).

**Table 5:** Diagnostic performance of AHI ≤6.0 mm vs. ultrasound (by observer and trial) Ultrasound reference standard: positive refers to full-thickness/complete or massive tear; negative refers to partial-thickness tear, tendinopathy, or no tear. Values are shown as % (95% CI) AHI: acromiohumeral interval; CI: confidence interval

Statistic	Obs 1 Trial 1	Obs 2 Trial 1	Obs 3 Trial 1	Obs 1 Trial 2	Obs 2 Trial 2	Obs 3 Trial 2
Sensitivity	39.29 (21.50-59.42)	32.14 (15.88-52.35)	53.57 (33.87-72.49)	42.86 (24.46-62.82)	32.14 (15.88-52.35)	53.57 (33.87-72.49)
Specificity	93.33 (81.73-98.60)	95.56 (84.85-99.46)	93.33 (81.73-98.60)	93.18 (81.34-98.57)	100.00 (92.13-100.00)	86.67 (73.21-94.95)
Accuracy	72.60 (60.91-82.39)	71.23 (59.45-81.23)	78.08 (66.86-86.92)	73.61 (61.90-83.30)	73.97 (62.38-83.55)	73.97 (62.38-83.55)

**Table 6:** Ultrasound prevalence and radiograph positivity rate (AHI ≤6.0 mm) Ultrasound prevalence refers to the proportion of cases classified as ultrasound-positive (full-thickness/complete or massive tear). Radiograph positivity rate is the proportion classified as AHI ≤6.0 mm by each observer and trial AHI: acromiohumeral interval

Measure	Ultrasound	Obs 1 Trial 1	Obs 2 Trial 1	Obs 3 Trial 1	Obs 1 Trial 2	Obs 2 Trial 2	Obs 3 Trial 2
Rate (%)	38	15	12	20	16	12	20

## Discussion

Principal findings

This service evaluation demonstrates that reporting radiographers can measure AHI on routine, nonstandardized AP shoulder radiographs with good to excellent intra- and interobserver reliability. Reliability remained high despite deliberate inclusion of poor radiographs representative of day-to-day acquisition variability, supporting the practicality of AHI measurement in routine reporting. However, the evaluation also identified specific circumstances in which measurement error can be clinically meaningful, including arm abduction and acromial variants/bony contour irregularities (e.g., unfused ossification centers).

Reliability in context and the value of SEM/MDC

Previous research has reported good reliability of AHI measurement, particularly when standardized true AP views are used, and poor-quality radiographs are excluded [16]. In contrast, Bernhardt et al. reported poorer agreement when measuring AHI on nonstandardized radiographs, highlighting the influence of positioning and projection [17]. The present evaluation bridges these contexts by assessing routine, nonstandardized radiographs without excluding technically challenging images and by focusing on the practical implications for reporting. Specifically, in addition to quantifying intra- and interobserver reliability, we evaluated the consequences of applying an AHI threshold (≤6.0 mm) as a binary “rule-in” feature for advanced RC tears using ultrasound as the reference standard.

Importantly, ICC alone can mask clinically meaningful differences because it is dimensionless and influenced by between-subject variability [26]. For this reason, SEM and MDC95 were calculated to express measurement error in millimeters. SEM values below 1 mm and MDC95 values of approximately 2 mm indicate that most repeated measurements fall within a narrow range; however, when AHI is interpreted using a fixed cutoff, even small differences may change classification close to the threshold. The observed threshold-crossing discrepancy (5.36 mm in one case; Figure 2) illustrates why landmark guidance should be explicit and why readers should interpret values near the cutoff with caution. This aligns with recommendations to supplement ICC with measurement error statistics when reliability findings may inform clinical decisions [26].

Sources of disagreement: arm abduction, beam orientation, and anatomy

**Figure 3:**
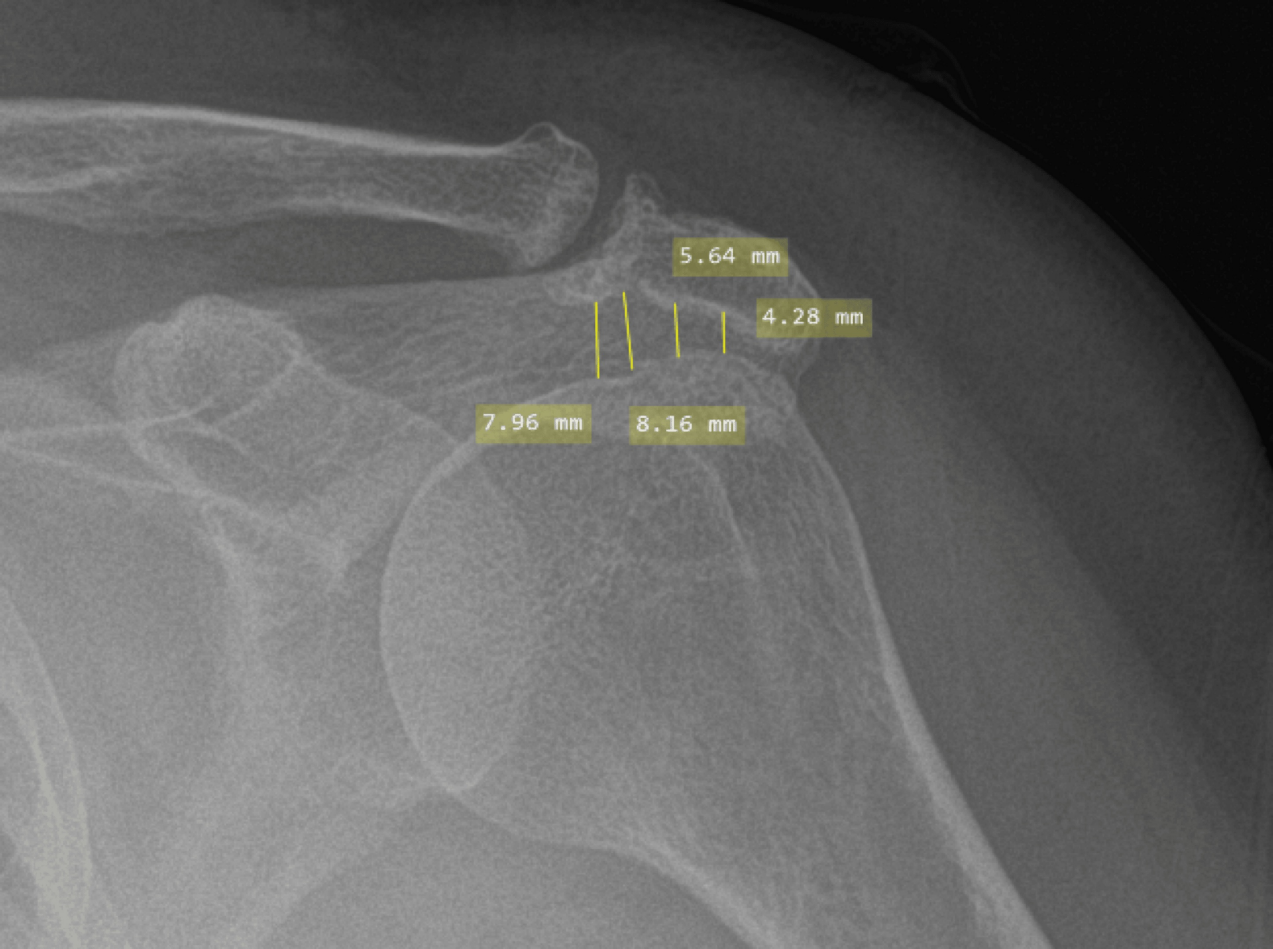
Illustrative effect of arm abduction and landmark choice on AHI measurement Illustrative example demonstrating how arm abduction/nonstandardized positioning can create multiple plausible inferior acromial-humeral head reference points and materially alter the measured AHI. Multiple example measurement locations are annotated on the same radiograph to show the potential range of AHI values that may result from landmark selection; these illustrative measurements were not performed by the study observers and were not included in the reliability analyses AHI: acromiohumeral interval

The largest between-observer discrepancies were concentrated in radiographs with arm abduction. Previous studies demonstrated that AHI decreases with increasing abduction and that beam orientation can change measured AHI by up to several millimeters [11,12]. In routine practice, abduction may reflect patient discomfort, limited mobility, or positioning variability. These factors cannot be eliminated entirely, but standardizing acquisition instructions (where feasible) and explicitly documenting when a radiograph is nondiagnostic for AHI measurement may reduce misclassification. To illustrate how abducted-arm positioning can create multiple plausible inferior acromial reference points and materially alter the measured AHI, we provide an annotated example (Figure 3); these illustrative measurements were not performed by the observers and were not included in the reliability analyses.

Anatomical variants and bony contour irregularities can also contribute to disagreement. In this evaluation, these features contributed to intraobserver discrepancies when different inferior acromial reference points were selected as the measurement landmark. Unfused acromial ossification centers (os acromiale) are a recognized variant and can alter the apparent inferior acromial margin on radiographs; awareness of such variants and careful landmark selection may reduce inconsistency [28]. A brief measurement guide with annotated examples (including abducted-arm projections and acromial variants) would likely reduce landmark inconsistency in training. For example, guidance could specify measuring to the most lateral continuous inferior acromial cortex and avoiding adjacent bony projections/ossicle margins as the inferior reference point, and could define when images are nondiagnostic for threshold-based interpretation due to marked abduction/rotation. Applied to the largest discrepancy case (Figure 2), this would likely reduce threshold-crossing variation by directing observers to a consistent inferior reference point, and in abducted-arm projections (Figure 3), it would reduce variability by discouraging threshold classification when positioning creates multiple plausible landmarks.

Hamada classification: why agreement is lower?

Hamada grading was recorded to reflect routine communication with orthopedics and to explore agreement for a commonly used grading system of cuff tear arthropathy [9,10]. In this evaluation, agreement was lower than for AHI measurement alone. This is expected because Hamada incorporates multiple features beyond AHI (e.g., acetabularization and glenohumeral osteoarthritis), and these qualitative features are more vulnerable to projectional differences on nonstandardized radiographs [10].

The discrepancy between high ICC for continuous AHI and lower kappa for Hamada underscores an important methodological point: converting continuous measurements to categories reduces information and can reduce apparent agreement, especially when values cluster around a cutoff [20,27]. In routine reporting, therefore, a structured narrative statement (e.g., “AHI appears reduced to ≤6.0 mm”) may be more reproducible than assigning a specific Hamada subgrade, unless standardized imaging and training are implemented.

Diagnostic performance and SPIN

The diagnostic results showed low sensitivity and high specificity for AHI ≤6.0 mm. This pattern mirrors prior work: Weiner and Macnab reported that a reduced AHI was uncommon in normal shoulders but present in only about half of confirmed tears, yielding high specificity but modest sensitivity [7]. Razmjou et al. and Kolk et al. similarly report low-to-moderate sensitivity with high specificity when using AHI thresholds to indicate full-thickness tears [4,19]. This high specificity/low sensitivity pattern is expected because AHI reduction reflects late-stage cuff failure [4,8,19].

High specificity supports using AHI ≤6.0 mm as a rule-in finding, consistent with the SPIN concept (a Specific test, when Positive, rules IN disease) [29]. In practical terms, when reporting radiographers identify a clearly reduced AHI on a routine AP radiograph, the likelihood of advanced RC pathology is high and false positives are uncommon. Conversely, because sensitivity is low, a normal AHI should not be used to rule out disease, and clinical assessment and response to conservative management should continue to guide escalation, consistent with CPG recommendations [1,5,6,13-15].

Why sensitivity is low: partial tears and disease stage?

Radiographs do not visualize tendon integrity directly. AHI reduction reflects superior humeral head migration, which is typically a late-stage consequence of chronic RC failure. Partial-thickness tears and many nonmassive full-thickness tears (e.g., <3 cm or isolated single-tendon tears) may preserve sufficient force coupling to prevent migration; therefore, AHI can remain within the normal range despite a tear, limiting sensitivity even when measurement reliability is high [4,8,19]. Systematic reviews also indicate that radiographic AHI is less reliable for detecting earlier stage disease than ultrasound or MRI, which directly assess tendon integrity [18,30].

Predictive values, prevalence, and pathway implications

PPV and NPV depend on disease prevalence in the population being tested [19]. In this pathway-selected cohort, PPV (79%) exceeded NPV (72%), consistent with a high-specificity test applied in a population with clinically suspected disease. However, the NPV remains limited because a negative test cannot exclude tears when sensitivity is low. This emphasizes that AHI should be interpreted as confirmatory rather than as a screening tool. In a service context with long ultrasound waiting lists, reliable identification of markedly reduced AHI could support more efficient triage by prioritizing patients with likely advanced pathology for specialist assessment, while maintaining ultrasound access for patients with persistent symptoms and nondiagnostic radiographs.

However, any pathway refinement should be undertaken cautiously. Before using AHI to alter referral pathways, services should implement standardized measurement guidance, identify when radiographs are nondiagnostic for AHI, and evaluate impact prospectively. The present findings confirm and contextualize existing evidence in routine radiographer reporting practice rather than replacing established diagnostic approaches.

Future work

Future service development could include a brief standardized AHI measurement guide with annotated examples illustrating common pitfalls (arm abduction, acromial variants, and landmark selection). Future guidance could specify a single inferior acromial reference rule (e.g., use the most lateral continuous inferior acromial cortex and avoid adjacent osseous projections/ossicle margins) and define when images are nondiagnostic for AHI due to abduction/rotation. A repeat evaluation after implementation could assess whether threshold-crossing discrepancies are reduced. Prospective evaluation of pathway impact, including downstream imaging utilization and time to definitive management, would be valuable before adopting AHI-based triage changes.

Limitations

This evaluation was conducted within a single NHS Trust with three observers, which may limit generalizability. Although radiographs were acquired under routine clinical conditions across multiple Trust sites, we did not capture site-level radiographic equipment details; therefore, we cannot quantify the extent to which hardware or local technique contributed to measurement variability. Replication using radiographs from multiple institutions with differing acquisition practices would be an important next step to confirm generalizability. Ultrasound was used as the reference standard and is operator-dependent, but it reflects routine care and is supported by evidence showing comparable accuracy to MRI for full-thickness tears in many contexts [30]. Sonographer experience was not captured because the analysis relied on issued clinical reports. The deliberate inclusion of nonstandardized radiographs increases external validity but may reduce comparability with standardized-view studies. Finally, because radiographs do not reliably identify partial tears and tendinopathy, sensitivity was expected to be limited.

## Conclusions

This service evaluation shows that reporting radiographers can measure the AHI on routine, nonstandardized AP shoulder radiographs with good to excellent reliability. Although AHI is an indirect marker and is not expected to detect all clinically significant RC tears, a clearly reduced AHI (≤6.0 mm) supports a higher likelihood of advanced RC pathology and can be interpreted as a useful rule-in feature in routine reporting.

In practice, these findings support incorporating consistent landmark guidance and clear statements about when AHI measurement is limited by positioning or projection. Targeted local training focusing on common pitfalls such as arm abduction and acromial variants/bony contour irregularities that affect inferior landmark selection may further improve consistency. Any pathway changes that use AHI to support triage should be implemented cautiously and evaluated prospectively to ensure that they improve patient flow and clinical decision-making without unintended consequences.

## Appendices

Hamada classification summary

**Table 7:** Hamada classification summary (radiographic cuff tear arthropathy) A summary of the Hamada radiographic classification is provided for reader reference [9,10] AHI: acromiohumeral interval

Hamada grade	Radiographic features (summary)
1	AHI > 6 mm
2	AHI < 5 mm
3	AHI < 5 mm with acetabularisation of the coracoacromial arch
4A	Glenohumeral joint space narrowing without acetabularization
4B	Glenohumeral joint space narrowing with acetabularization
5	Humeral head collapse/osteonecrosis (end-stage arthropathy)
